# Contribution of Natural Inhibitors to the Understanding of the PI3K/PDK1/PKB Pathway in the Insulin-mediated Intracellular Signaling Cascade

**DOI:** 10.3390/ijms9112217

**Published:** 2008-11-12

**Authors:** Jae Youl Cho, Jongsun Park

**Affiliations:** 1 School of Bioscience and Biotechnology, and Institute of Bioscience and Biotechnology, Kangwon National University, Chuncheon 200-701, Korea. E-Mail: jaecho@kangwon.ac.kr; 2 Department of Pharmacology, Cell Signaling Laboratory, Daejeon Regional Cancer Center, Cancer Research Institute, Research Institute for Medical Sciences, College of Medicine, Chungnam National University, Taejeon, 301-131, South Korea

**Keywords:** Diabetes mellitus, phosphatidylinositol 3-kinase, 3-Phosphoinositide-dependent protein kinase-1, protein kinase B, natural inhibitors

## Abstract

The critical initial steps in insulin action include phosphorylation of adapter proteins and activation of phosphatidylinositol 3-kinase (PI3K). One of important components in this process is a protein called Akt/protein kinase B (PKB). The work of numerous different researchers indicates a role of PKB in regulating insulin-stimulated glucose uptake. The crucial role of lipid second messengers in PKB activation has been dissected through the use of the PI3K-specific inhibitors wortmannin and LY294002. Receptor-activated PI3K synthesizes the lipid second messenger PtdIns[3,4,5]-trisphosphate, leading to the recruitment of PKB to the membrane. Membrane attachment of PKB is mediated by its pleckstrin homology domain binding to PtdIns[3,4,5]-trisphosphate or PtdIns[3,4]-bisphosphate with high affinity. Activation of PKB alpha is then achieved at the plasma membrane by phosphorylation of Thr308 in the activation-loop of the kinase domain and Ser473 in the carboxy-terminal regulatory region, respectively. 3-Phosphoinositide-dependent protein kinase-1 (PDK1) is responsible for T308 phosphorylation. The usage of specific inhibitors and natural compound has significantly contributed to investigate the molecular mechanism of PI3K/PDK1/PKB signaling pathway, leading to the putative therapeutics benefits of patients. This review focuses on the contribution of natural inhibitor or compound in our understanding of the mechanism by which insulin induces, especially in PI3K/PDK1/PKB signaling.

## 1. Introduction

The discovery, production, and clinical use of insulin in the 1920s greatly prolonged the life expectancy of patients with insulin-dependent diabetes mellitus [1]. It is now established that diabetes mellitus is the most common cause of end-stage renal failure, blindness, and lower limb amputations in adults, and is a major risk factor for cardiovascular disease and stroke [2]. Today, diabetes is the most common metabolic disease in the world. Almost every day, 1,700 new cases of diabetes are diagnosed in the United States [3]. Normal glucose homeostasis requires the finely tuned orchestration of insulin secretion by pancreatic β cells in response to subtle changes in blood glucose levels, delicately balanced with secretion of counter-regulatory hormones such as glucagon. The importance of insulin in glucose homeostasis is emphasized in metabolic malfunctions in which insulin action is defective [4]. Autophosphorylation of the insulin receptor by insulin results in the recruitment and activation of intracellular downstream signaling molecules and leads to glucose uptake and various other biological effects [5]. A lack of insulin or insulin resistance, or defects in the insulin signaling pathways are the cause of metabolic diseases such as diabetes mellitus, which is characterized by hyperglycemia [6]. Therefore, to make better treatment of such relevant diseases, it is absolutely necessary for us to understand as to how intracellular signaling molecules can be activated by insulin action. Some natural products indeed greatly helped us to understand insulin-induced molecular events. In this review, we focused on PI3K/PDK1/PKB pathway, a major signaling part, triggered by insulin and its understanding by naturally occurring inhibitors derived from microorganisms.

## 2. Natural inhibitors targeting PI3K/PDK1/PKB pathway in insulin-mediated signaling events

At present, the treatment of diabetes mainly involves a sustained reduction in hyperglycemia by the use of biguanides, thiazolidinediones, sulphonylureas, D-phenylalanine derivatives, meglitinides and α-glucosidase inhibitors in addition to insulin. However, due to unwanted side effects the efficacies of these compounds are debatable and there is a demand for new compounds for the treatment of diabetes [7]. Natural products have been suggested as a rich, as yet unexplored source of potentially useful antidiabetic drugs. These compounds, produced by plant, bacteria and fungi, can be toxic to other organisms and have a variety of physiological effects in animals. Many pharmaceutical agents have been discovered by screening natural products from a wide range of microorganisms and event plants. For example, rapamycin (sirolimus) was originally discovered at Wyeth-Ayerst Pharmaceuticals in a screen for antifungal agents and later found to have potent immunosuppressive activity [8]. In addition, the natural products rapamycin, wortmannin, geldanamycin and okadaic acid have been found to possess various pharmacological effects such as immunosuppressive and anti-proliferative actions [9; 10]. Through numerous molecular studies, it has been demonstrated that their molecular targets are interestingly found at insulin signaling cascades.

### 2.1. Wortmannin: An inhibitor of PI-3-kinase family members

**Figure 1.**
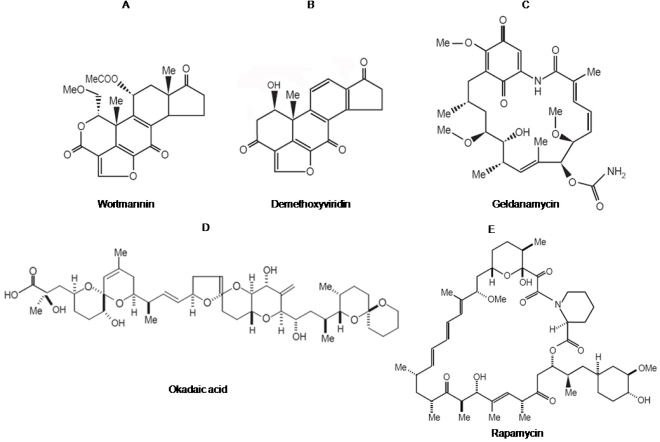
Molecular structures of natural products that inhibit insulin signaling.

Wortmannin is a hydrophobic estrogen-related fungal metabolite (Figure 1A) from the fungus *Talaromyces wortmanni* [11]. The *in vivo* anti-inflammatory and immunosuppressive effects shown by wortmannin first suggested that it was a potent inhibitor of signal transduction pathways [11]. Wortmannin blocks cellular responses emanating from stimulation of G-protein-coupled receptors. For example, wortmannin inhibits stimulation of neutrophils [12], histamine secretion by basophilic leukemia cells [13] and nitric-oxide production in macrophages [14].

**Figure 2.**
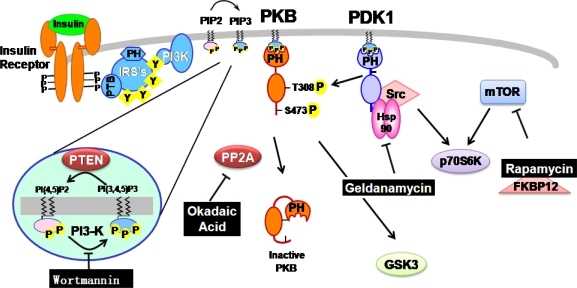
Inhibition of each signaling molecules by natural products in insulin signaling.

In mammalian cells, several lines of evidence indicate that the growth-factor-activated PI-3 kinase (PI3K) is potently inhibited by wortmannin, which was originally isolated from soil bacteria [15]. PI3K was originally identified as the protein responsible for the phosphorylation of the D-3 position on the inositol head group of phosphoinositides (Figure 2) [16]. There is growing evidence to suggest that insulin stimulation of PI3K is essential for insulin’s regulation of metabolism. This conclusion is largely based on studies using two structurally independent PI3K inhibitors, wortmannin and LY294002. These inhibitors block insulin stimulation of a number of metabolically important responses including the antilipolytic effect [17], activation of acetyl CoA carboxylase [18] and induction of membrane ruffling [19]. Importantly for insulin’s regulation of glucose metabolism it has been found that wortmannin blocks activation of glycogen synthase [20]. Wortmannin also blocks insulin stimulation of signaling intermediates such as the 70 kDa ribosomal protein S6-kinase (p70S6K) [21], Protein kinase B (PKB) [22] and in parallel activates glycogen synthase kinase-3 (GSK-3) [23]. These studies place PKB upstream of GSK-3 activation and also upstream of p70S6K activation (Figure 2). This is important as both GSK-3 and p70S6K have been implicated as elements of insulin signaling pathways leading to glycogen synthase activation. Therefore these studies begin to suggest the layout of signaling pathways linking the insulin receptor with regulation of glycogen synthase.

Wortmannin also inhibits the antigen-dependent stimulation of PI3K activity in basophils [24] as well as stimulated PtdIns[3,4,5]-trisphosphate production in neutrophils [25], consistent with a block in PtdIns[4,5]-bisphosphate phosphorylation by PI3K; purified p110-p85 PI3K is potently inhibited by wortmannin *in vitro* [26]. Furthermore, studies with anti-wortmannin antibodies and site-directed mutagenesis reveal that wortmannin forms a covalent complex with an active-site residue of bovine PI3K, lysine 802 of the 110 kDa catalytic subunit [27]. This active-site lysine residue is essential for PI3K activity and is well conserved throughout all members of the PI3K-related protein family.

Although wortmannin potently inhibits the PI3-kinase with a 50% inhibitory concentration (IC_50_) of 5 nM, more recent studies have shown that it also inhibits PI4-kinases. A wortmannin-sensitive membrane-associated PI4-kinase was identified and cloned in mammalian cells [28]. Demethoxyviridin, a structural analog of wortmannin, inhibits an unidentified membrane-associated PI4-kinase from the fission yeast *Schizosaccharomyces pombe* (IC_50_ = 100 nM) [29]. Interestingly, wortmannin is also toxic to the budding yeast *S. cerevisiae*. However, although wortmannin can inhibit the yeast PI3-kinase VPS34 *in vitro* at concentrations higher (IC50 = 3 μM) than those required to inhibit the mammalian PI3-kinase [30], mutant yeast cells lacking VPS34 are viable and remain wortmannin sensitive [31]. This result suggests that wortmannin toxicity in yeast is mediated via another target. These observations led to the identification of a wortmannin target in yeast as the PI4-kinase STT4 [31]. Thus, overexpression of STT4 in yeast rescues cells from wortmannin toxicity. Moreover, STT4 PI4-kinase activity *in vitro* is sensitive to 10 nM wortmannin. The inhibitory activity of wortmannin is not restricted to PI3- and PI4-kinases, and at higher concentrations wortmannin also inhibits several members of a novel family of PI-related protein kinases. These wortmannin-sensitive enzymes include the mammalian target of rapamycin mTOR (IC_50_ = ~200 nM) [32] and yeast TOR1 (IC_50_ = ~100 to 200 nM) [33], and also DNA damage control proteins including the human DNA-dependent protein kinase (DNA-PK) (IC_50_ = 16 nM]) [34], the ataxia-telangiectaxia (AT) mutated (ATM) protein (IC_50_ = 150 nM), and the ATM and Rad3-related protein ATR (IC_50_ = 1.8 μM) [34, 35].

### 2.2. Geldanamycin: An inhibitor of Hsp90 and of Hsp90-dependent-signalling components

Geldanamycin is a benzoquinone ansamycin (Figure 1C) natural-fermentation product that was originally thought to be a direct tyrosine-protein-kinase inhibitor. However, subsequent studies revealed that geldanamycin and two other structurally related analogues (herbimycin and macbecin) bind to and inhibit the functional role of 90 kDa heat-shock protein (Hsp90) instead [36]. Together with Hsp70 and a variety of other associated chaperones, including p60, p23, and immunophilins (cyclophilin 40 and FKBP52 or FKBP54), Hsp90 was highly conserved and played an important role in refolding certain denatured proteins under stress conditions. Unlike the more general Hsp70 and Hsp60 chaperones, Hsp90 appeared to have substrate-specific folding activity. Hsp90 has an additional role in the conformational regulation of certain signal transduction molecules. These include oncogenic kinases (e.g. v-Src and PKB) and a variety of members of the steroid-receptor family. Geldanamycin thus interferes with the activity of oncogenic kinases and steroid receptors by targeting their unique Hsp90-dependent function. Hsp90 functions as part of a multi-chaperone complex, involving the dynamic association with various accessory co-chaperones and client proteins [37]. Binding and hydrolysis of ATP are critical for the operation of a chaperone 'cycle', which involves a complex series of loading and unloading events that are essential for client protein stabilization and function. The ATP binding state of Hsp90 determines combinations of co-chaperones to bind at particular stages of the chaperone-client protein cycle. In particular, formation of a ‘mature’ Hsp90 complex is required for client protein function and stability in an ATP-dependent manner. Geldanamycin docks in the ATP binding site located in the activity [38]. Consequently, the formation of mature Hsp90 complex and intrinsic ATPase activity cannot be occurred.

Recently, the components of PI3K signaling pathway, such as PKB [39] and 3-Phosphoinositide-dependent protein kinase-1 (PDK1) [40] are also suggested to be a client protein for Hsp90 in HEK 293 cell and endothelial cells (Figure 2). It has been shown that Hsp90 can directly bind and stabilize PKB activity by preventing PP2A-mediated dephosphorylation [39]. Hsp90 can also recruit PKB to the eNOS complex during NO-dependent angiogenic processes [41]. Furthermore, it has also been suggested that Hsp90 acts on PDK1 in a similar manner to PKB [39, 40]. However, the mode of Hsp90’s action on PKB and PDK1 is somewhat different. Hsp90 inhibitors did not inhibit PKB-Hsp90 binding or PKB activity itself [39; 42], whereas Hsp90 inhibitors are able to suppress PDK1-Hsp90 complex formation, leading to PDK1 destabilization without directly inhibiting PDK1 activity [K, Yang and J, Park - unpublished data, [43]. The progress of geldanamycin into the clinic was stopped due to instability and the unacceptable hepatotoxicity seen at therapeutic doses during preclinical *in vivo* studies [44]. Further analogues were developed for clinical use, which included 17-AAG [45].

Once insulin binds to insulin receptor, the receptor gets dimerized and trans-phosphorylated in several tyrosine residues of the intracellular domain. This phospho-tyrosine acts as a docking site for insulin receptor substrate-1 (IRS1). IRS1 also is phosphorylated and recruits to the PI3K. The activated PI3K generates PtdIns[3,4,5]P3 from PtdIns[4,5]P2 on the plasma membrane. PKB and PDK1 then are recruited to plasma membrane wherein PDK1 activates PKB as well as p70S6K. Wortmannin inhibits the activity of PI3K which produces the PtdIns[3,4,5]-trisphosphate, PIP3, while okadaic acid blocks the activity of PP2A which is key serine/threonine phosphatase. Recently, it has been reported that Hsp90 is involved in PDK1 activation. Geldanamycin is also known to regulate the interaction between Hsp90 and PDK1 but not PKB in this process, although long exposure of this compound lowered PKB activity in the cells. Independently, FKBP12-rapamycin complex inhibits mTOR, leading to the inhibition of S6K.

### 2.3. Okadaic acid : An inhibitor of serine/threonine protein phosphatase

Okadaic acid is a marine natural toxin product (Figure 1D) that originally isolated from extracts of the marine sponge *Halichondria okadai* as a potential anti-cancer agent [46]. Okadaic acid was subsequently found to have cancerous tumor promoting activity in the two-stage model of carcinogenesis on mouse skin [47], which makes a limitation in its clinical use. Moreover, these seemingly paradoxical responses to okadaic acid exposure have subsequently led to the widespread recognition of the central roles that several classes of protein serine/threonine phosphatases, the primary cellular targets of okadaic acid, play in the regulation of many essential cellular processes, including metabolism, growth, division, and death [48]. Hence, okadaic acid has emerged as a key laboratory tool for identifying and studying the myriad of events associated with the inhibition of protein serine/threonine phosphatases [49; 50]. Okadaic acid is the first member of an entire class of remarkably distinct secondary metabolites from such disparate organisms as bacteria, blue-green algae, red algae, and even insects that together comprise the "okadaic acid class" of phosphatase inhibitors [51, 52].

Today, okadaic acid is being employed in basic studies directed towards understanding such diverse human disease related processes as cancer, AIDS, inflammation, osteoporosis, Alzheimer's, and diabetes [49; 50]. Phosphorylation level in cells results of a delicate balance between protein phosphatases and protein kinases. Kinases transfer a phosphate from ATP to a protein. Phosphatases remove the phosphate group from the substrate protein [50]. Regulation of the levels of phosphorylated proteins is fundamental to a large number of cellular processes. These include changes in gene expression, muscle contraction, protein synthesis, intracellular transport and cell cycle progression, apoptosis or glycogen metabolism. Breakdown of endogenous glycogen stores provided glucose, which is a fundamental source of energy for all eukaryotic cells. These energy stores are replenished from glucose in the diet [53]. Certain tissues such as gut, muscle and adipose tissue, have acquired a highly specialized glucose-transport systems. The activity of transporters can be rapidly upregulated to allow these tissues to increase their rate of glucose transport by 10–40-fold in minutes. Those systems are crucial during the absorptive period (after a meal), to facilitate the rapid insulin-dependent storage of glucose in muscle and adipose tissue, so preventing large fluctuations in blood glucose levels. Dysfunctional glucose uptake into muscle and fat cells contributes to the onset of type II diabetes [53].

However, the application of okadaic acid is presently limited by several factors. Firstly, this compound is an additional non-phorbol-12-tetradecanoate-13-acetate-type tumor promoter, which was never considered as a treatment option [9]. Secondly, okadaic acid lacks sufficient specificity in its inhibition of okadaic acid-sensitive protein phosphatases. Hence, relatively indiscriminant phosphatase inhibition may simultaneously affect a variety of important cellular processes, in addition to the targeted ones. Finally, the current commercial sources of okadaic acid are primarily isolation in small quantities from cultured dinoflagellates, single celled marine microorganisms [54]. This provides research quantities at high costs, whereas the availability of specific inhibitors of the protein serine/threonine phosphatases remains to be developed. However, alternative sources of okadaic acid and, more importantly, structurally related compounds have been emerging in recent years via laboratory total synthesis [55]. Total synthesis offers unique opportunities to tailor the details of the okadaic acid architecture with the aim of developing an empirical understanding of the relationship between structure, function, and specificity [56]. Concurrently, structural information of the protein phosphatase targets of okadaic acid has been acquired, largely through X-ray crystallography [57]. The detailed topological and intermolecular recognition data of the okadaic acid receptors combines with the ability to generate specifically designed structural variants of okadaic acid to provide unique contemporary opportunities to develop specific regulators of essential cellular processes via the selective modulation of phosphatase activity.

### 2.4. Rapamycin: an inhibitor of mTOR-dependent signaling pathways

Rapamycin (Figure 1E) is a natural product with antimicrobial and immunosuppressant activities due to its ability to inhibit signal transduction cascades [58]. Rapamycin is a potent immunosuppressant that inhibits interleukin-2 (IL-2) signaling and prevents T-cell proliferation by inhibiting progression from G1 to S-phase of the cell-cycle. The fact that IL-2-deficient mice are not markedly immunocompromised, even though rapamycin is potently immunosuppressive, suggests that rapamycin probably blocks signaling by additional cytokines, possibly including interleukins 4, 7, 9 and 13, all of whose receptors share the gamma-subunit of the IL-2 receptor. Rapamycin may therefore inhibit a downstream signaling component shared by these receptors. In both yeast and mammalian cells, the action of rapamycin is mediated by its association with a highly conserved binding protein, the peptidylprolyl isomerase FKBP12 [59]. Remarkably, yeast mutants lacking FKBP12 are viable and resistant to rapamycin toxicity, indicating that both the protein and the drug are required for rapamycin action and providing strong support for a model in which the FKBP12-rapamycin complex is the active *in vivo* agent. Overexpression of FKBP12 in mammalian cells increases sensitivity to rapamycin, and cell lines with reduced levels of FKBP12 are rapamycin resistant, providing evidence that FKBP12 is the conserved target of rapamycin action in yeast and mammals [60].

The targets of the toxic FKBP12-rapamycin complex are novel kinase homologs, the target-of-rapamycin kinase 1 (TOR1) and TOR2 proteins in yeast and mTOR protein in mammals, which are conserved from yeast to humans. Genetic studies in yeast, in which rapamycin-resistant mutants were isolated, first implicated the TOR1 and TOR2 gene products as targets of the FKBP12-rapamycin complex [61]. Analysis of the cloned TOR1 and TOR2 genes revealed the potential to encode large (~ 280-kDa) proteins that have 67% overall identity [62]. However, despite this remarkable similarity, TOR1 and TOR2 serve both shared and distinct functions. Deletion of the TOR1 gene confers only a modest growth defect under most conditions, whereas deletion of TOR2 is lethal. Subsequently, a mammalian TOR homolog (mTOR/ FRAP/ RAFT1/ SEP/ RAPT1) was identified by its ability to physically interact with FKBP12-rapamycin and was found to have ~50% identity to the yeast TOR proteins [63]. The highest level of identity between the yeast and the mammalian TOR proteins is in the carboxyl-terminal domain, which exhibits sequence identity to both lipid and protein kinases. The X-ray structure of FKBP12-rapamycin bound to a small portion of the mTOR protein (the FRB domain) has been solved [64]. This structure revealed that rapamycin docks into a hydrophobic pocket on the surface of the TOR protein that has been highly conserved from yeast to humans. Few protein-protein contacts are apparent in the X-ray structure, but they should exist and play an important role in the complex, because rapamycin does not bind to the mTOR protein alone and thus FKBP12-TOR contacts should contribute to the ternary complex. Genetic studies had identified three residues that play a critical role in FKBP12-rapamycin binding to the yeast TOR1 and TOR2 proteins [65]; these three residues, Ser1975, Trp2042, and Phe2049, are all conserved in the mammalian TOR protein and form the base and sides of the hydrophobic rapamycin binding pocket on mTOR [66].

It has been a question that what are the substrates of mTOR and which of them are involved in cell-cycle control and translational regulation. Several studies have demonstrated that rapamycin inhibits the activation of p70S6K, but this kinase does not appear to be a direct target for rapamycin. The S6 protein is a component of the 40S ribosomal subunit and, as phosphorylation of the S6 protein by p70S6K correlates with increased translation, this suggests one possible mechanism by which rapamycin could inhibit translation. On the other hand, p70S6K has also been linked to transcriptional control by the cAMP-responsive element modulator, suggesting a role in transcriptional regulation [67]. It is worth noting that the effects of rapamycin on yeast and mammalian cells are remarkably similar, but no yeast homologue of p70S6K is apparent in the now-completed sequence of the yeast genome. Another downstream target of mTOR is the eukaryotic translation-initiation factor 4E (eIF-4E)-binding protein PHAS-I, which plays a key role in regulating translational initiation. PHAS-I binds to and inhibits the function of eIF-4e, which normally recognizes the 7-methylguanine cap on mRNA to initiate translation. PHAS-I is phosphorylated and inactivated in response to growth-factor stimulation, releasing eIF-4e to activate translation. Rapamycin blocks the phosphorylation of PHAS-I *in vivo* and inhibits mTOR-dependent phosphorylation of recombinant PHAS-I *in vitro* [68]. Recent findings suggest that the two proteins known to function downstream of mammalian mTOR (p70S6K and PHAS-I) may represent two distinct signaling pathways, because the phosphorylation of PHAS-I by mTOR is still rapamycin sensitive in cells expressing a rapamycin-resistant form of p70S6K [69]. The cyclin-dependent-kinase (CDK) inhibitor p27kip1 also plays a role in rapamycin-dependent cell cycle arrest in mammalian cells. p27kip1 is degraded following stimulation of T cells with IL-2, and rapamycin treatment prevents p27kip1 degradation [70]. In addition, cell lines that are partially resistant to rapamycin express very low levels of p27kip1, and cells derived from p27-knockout mice are also partially rapamycin resistant [71]. The regulation of p27kip1 may therefore represent a rapamycin-sensitive step in pathways regulating cell-cycle progression and arrest in T cells.

## 3. Conclusions

These five natural products have significantly contributed the understanding of molecular mechanisms of action in PI3K/PDK1/PKB pathways activated by insulin and other growth factors. Toxins that originally evolved to kill competing microorganisms can be used as quite specific agents in complex animals, although these are known to be toxic in our body. In particular, toxin compounds mentioned in this review are reported to be cancer promoter or toxic in cellular or organic systems and mainly known as a blocker of insulin effects. Due to this, these toxins can never be used to human for curing insulin-mediated diseases such as diabetes. Nonetheless, these compounds greatly helped in exploring molecular activation mechanisms on the functional activation of PI3K, PDK1 and PKB, as well as their substrate proteins upon insulin treatment. These finding also rendered us to develop novel drugs to treat insulin-dependent diabetes and other metabolic diseases. These aspects indicate that most of natural products including toxins can be considered as a valuable source to investigate cellular events, regardless of their toxicological profiles. Therefore, it is expected that additional biochemical events will be further understood by another natural inhibitors derived from microorganisms and plants and will give us an opportunity to realize more complicate biological processes.
